# The Society of European Robotic Gynaecological Surgery (SERGS) Pilot Curriculum for robot assisted gynecological surgery

**DOI:** 10.1007/s00404-017-4612-5

**Published:** 2017-12-13

**Authors:** Peter Rusch, Rainer Kimmig, Fabrice Lecuru, Jan Persson, Jordi Ponce, Michel Degueldre, René Verheijen

**Affiliations:** 1 Department of Obstetrics and Gynecology, University Hospital Duisburg-Essen, Hufelandstr. 55, 45147 Essen, Germany; 2 Sorbonne Paris Cité, Faculté de Médecine, Université Paris Descartes, Paris, France; 3 Assistance Publique-Hôpitaux de Paris, Hôpital Européen Georges-Pompidou, Chirurgie Cancérologique Gynécologique et du Sein, Paris, France; 4 Department of Obstetrics and Gynecology, Skane University Hospital and Lund University, Lund, Sweden; 5 Department of Obstetrics and Gynecology, Universitat da Barcelona, Barcelona, Spain; 6 European Robotic and Minimal Invasive Surgery Institute (ORSI) cvba, Melle, Belgium; 7 Department of Gynaecological Oncology, University Medical Center, Utrecht, Netherlands

**Keywords:** Educational program, Robotic, Fellowship, Training

## Abstract

**Purpose:**

To set forth experiences in the context of the SERGS Pilot Curriculum—the first standardized educational program for robotic use in gynecological surgery—in terms of feasibility, effectiveness and potential for certification.

**Methods:**

The Society of European Robotic Gynecological Surgery (SERGS) outlined a Pilot Curriculum for standardized education in robot-assisted laparoscopic gynecological surgery. Its feasibility and acceptance were checked in the form of a fellowship pilot program conducted at four European Centers of Excellence for robot-assisted surgery. Results and conclusions derived from this pilot program are presented.

**Results:**

The SERGS Pilot Curriculum defines criteria for a standardized training and assessment of performance, boosts the learning curve of the candidate and increases contentment at work. Regarding face validity, it proves valuable as finally all candidates could perform the outlined procedure safely and efficiently without supervision.

**Conclusion:**

Due to the immense increase of robotic procedures in gynecology standardized training curricula are indispensable. This seems highly necessary to ensure patients’ safety and surgical outcome. The SERGS Pilot Curriculum sets standards for a stepwise theoretical and practical training in gynecological robotic procedures. It seems feasible as instrument for accreditation as gynecologic robotic surgeon. Though as a general applicable guideline for systematic training in robot-assisted surgery, a definite curriculum should have a more definite timeline and implementation of a structured assessment of performance.

**Electronic supplementary material:**

The online version of this article (10.1007/s00404-017-4612-5) contains supplementary material, which is available to authorized users.

## Introduction

**Fig. 1:**
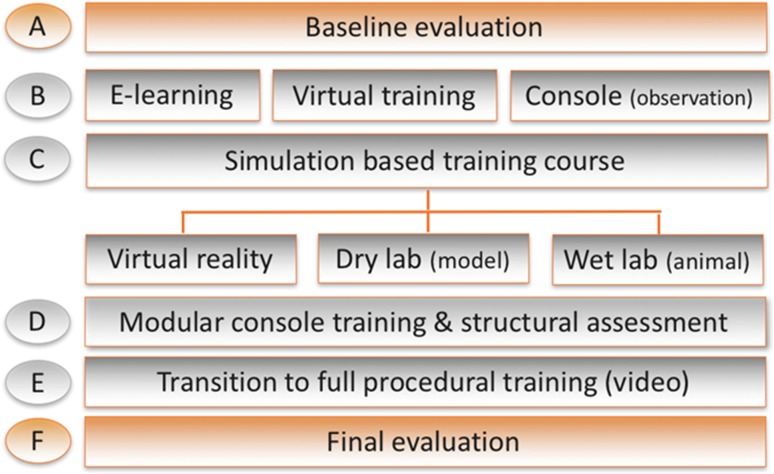
Modular training program (adapted from the ERUS curriculum [15])

The introduction of robotic devices in surgery has opened new options regarding complexity and outcome of surgery. Especially radical oncological surgery [1]. Complex radical oncological procedures can now easily and safely be performed laparoscopically, even in obese patients [2, 3]. The number and types of robotic devices is increasing, thus far only the DaVinci^®^ (Intuitive Surgical Industries, Sunnyvale, California, USA) has been marketed extensively. These multifunctional systems clearly warrant special training to be versed in its use and to make full and safe use of all features. In this context, literature concerning education in robotic surgery states the need for formal standardized curricula, but existing guidelines up to date only describe training in broad terms [4–6]. With a growing number of guidance documents in gynecology and in related surgical fields, professional bodies are still working on well-defined and definitive regulative educational programs. To date only the European Association of Urology (EAU) has managed to draft a validated curriculum for modular training and stepwise education of robot-assisted urologic procedures. This program is planned in the form of a comprehensive multi-step scheme with three educational key components: (1) e-learning (technical features, clinical indications, regulatory issues) and bedside console teaching, (2) an intensive structured training on virtual simulators, live and cadaver models, and (3) supervised modular procedural training (see Fig. 1). In contrast to other existing programs, only this EAU Robotic Urology Section (ERUS) program encompasses the whole learning path, from technical instruction to patient procedures. This 12-week program ends with a final skills evaluation of the ability to perform the most common robot-assisted procedure in urology, the radical prostatectomy (RARP). This is tested by blind revision of a video-documented full procedure under use of previously validated global assessment scores for technical skills (GEARS) and non-technical skills (NOTSS) by their mentors. The Society of European Robotic Surgery (SERGS) based their pilot program on the results of the ERUS curriculum.

## Methods

In 2015 SERGS outlined a Pilot Curriculum for standardized education of robotic use in gynecological surgery. The final aim of this program was to test the feasibility and standardize an educational program, to verify if it was effective itself in evaluating a candidate’s surgical performance and to assess its potential for use as an instrument for certification.

The SERGS pilot curriculum was planned in the form of a fellowship program and aimed to increase robot-assisted surgical skills of selected fellows in moderate complex procedures. Hysterectomy and pelvic lymphadenectomy were chosen as index procedures as these procedures would commonly and basically form part of radical oncological surgery.

After open invitation to all its members SERGS in May 2015 invited four large centers of excellence for robot-assisted surgery to participate and train as well as propose fellows. These centers had to fulfill eligibility criteria (see Supplementary Data, Tab. 1) including i.a. an established robotic team with an adequate workload to prove experience. Leaders of the elected centers were invited to nominate trainees whom they regarded as suitable candidates for the fellowship. Applicants should be certified gynecologists, but novices in robotic surgery, i.e. with little or no robotic console experience. They completed an initial survey detailing demographic and training-related information and submitted a letter of motivation.

The tri-modular course of the curriculum followed a validated and published format [7] (see Supplementary Data, Tab. 2). Fellows could start training at any time in their dedicated training center but should consecutively pass the various modules of the curriculum. The Curriculum outlined the use of different assessment tools for evaluation of progress. For technical skills, the GEARS or the Objective Structured Assessment of Technical Skills (OSATS) scale were suggested. Both instruments facilitate a quick evaluation and reference to earlier performance to measure progress, while only the GEARS scale was specifically designed and validated for robot-assisted surgery [8, 9]. The NOTSS scale was outlined as a short instrument to integrate also non-technical competencies [10, 11].

The first section of the curriculum included didactic and system training at the home education center. This part was planned for about 1 month and—in the sense of a modular training—should have been completed before part 2 started with a 1-week procedural training at an European education center for robotic surgery. The third part focused on in-house training as mentored work. It was planned for about 6 months. A portfolio was planned to be subsequently built in the home institution with modular training. Finally, formal approval of a completed logbook and assessment of a video-case recording by a SERGS expert were planned to lead to certification as robotic gynecological surgeon.

### Part 1: didactic part and virtual training (in-house)

Didactic introduction into correct docking, use of instruments and solving technical problems was done by the fellows’ dedicated supervisor or by a representative of Intuitive Surgical Systems (ISI) in the function of a system instructor. Didactic knowledge of fellows was tested using online test modules offered by the manufacturer or theoretical teaching from their dedicated supervisor. Virtual training in advance of the dry-lab section was not mandatory, as it depended on availability at the host institute. Nevertheless, fellows were encouraged to perform at least a number of virtual exercises. Attestations of didactic and virtual training had to be sent to the SERGS educational committee before start of the 1-week dry-and wet-lab section.

### Part 2: dry- and wet-lab training

An important and vital part of the educational program was a 5 days hands-on intensive training in a dedicated and fully equipped training center (European Robotic and minimal invasive Surgery Institute, ORSI, Ghent, Belgium). When the American Food and Drug Administration (FDA) approved the DaVinci-Robot, the company was required to provide comprehensive training on the device. Among more than 24 educational centers located all over the world, the independent ORSI training center is one of three of such centers in Europe.

An introduction was provided with scientific results about differences between the classic laparoscopic approach and the particularities of robotic surgery.

Virtual exercises were performed on a DaVinci training console (DVSS) equipped with Mimic^®^ training software (Mimic Technologies, Inc., Seattle, WA 98104, USA), that uses comprehensive metrics and experienced surgeon data in the MScore^®^. The software has been tested for face [12], construct [12, 13] and predicative validity [14]. Thus, this virtual training system resembles the real-life situation, is indeed discriminatory, i.e. measures the ability tested for, and makes estimates with regard to the future performance of the candidate.

A “meet-the-expert”-session with video presentation of complex robotic procedures highlighted the spectrum of robot-assisted surgery.

Finally, in the wet-lab training fellows had the option to operate on live anesthetized pigs and on cadaver models exercising suture techniques, knotting, performing hysterectomies, adnexectomies and finally pelvic and para-aortic lymphadenectomies under the supervision of the expert on site. The curriculum scheduled passing a virtual training of standardized skill tasks initially and at the end of the week to elucidate their progress of robotic skills throughout the intensive training of the week.

### Part 3: procedural training (in-house)

Third part of the tri-modular curriculum started with “real-life” in-house training in the host institute. Fellows had to translate their improved theoretical and practical knowledge into the daily robotic routine. Depending on their individual skills from the perspective of their designated proctor, they were held to perform moderate to complex gynecological procedures per the schedule of the curriculum.

To take stock of the progress in every fellow’s robotic performance and to discuss feasibility of the program 2 telephone video conferences were held after 6 and 12 months with fellows and tutors, moderated by the chairmen of SERGS’s Educational Committee. Interim-results were presented at the SERGS annual conference at Barcelona after 9 months.

At the first tele-conference, fellows’ experiences so far were discussed especially in terms of supervision by the designated in-house proctors. A second telephone video session was held to consider the results of the curriculum from both the fellows’ and the supervisors’ perspective and to draw a conclusion in the context of feasibility of the pilot educational program.

A video-documented hysterectomy had to be assessed by one of the tutors from another center than the fellow’s center, who served as external reviewer. This evaluation together with the completed logbook had to be sent to the SERGS educational committee for final approval.

## Results

Four fellows altogether were elected to take part in the SERGS Pilot Curriculum for education in robotic gynecological surgery. Their level of surgical experience ranged from junior resident to staff surgeon (see Supplementary Data, Tab. 3).

In the first part of the curriculum all candidates passed a didactic in-house training by online test modules and under supervision of their designated in-house proctors or by a host-instructor of the manufacturer. At the end of part 1, they were all trained on the system, particularly on correct trocar placement and docking, insertion/exchange of instruments and troubleshooting of common technical problems. Two candidates gained experience on the virtual reality DVSS during this part of the curriculum, but none had clinical robotic experience.

Dry- and wet-lab training in the second part of the curriculum took part in a 5-day course at the ORSI education center for robotic surgery. All fellows showed improvement over the week regarding their overall score on the DVSS virtual module (Table 1). Some candidates started with high scores already with only marginal improvement, while others showed much steeper graphs due to lower starting scores.

**Table 1:** Examples of exercises and assessment of performance (overall score, %) at baseline and after 1-week training at ORSI, Ghent

Candidate	1		2		3		4	
Time 0 = baseline 1 = after 5 days training	0 (%)	1 (%)	0 (%)	1 (%)	0 (%)	1 (%)	0 (%)	1 (%)
Exercise								
Endo-wrist manipulation: “Matchboard 2”	74	72	84	94	56	70	56	69
Energy and dissection: “Energy Switch 2”	80	99	97	97	74	89	71	93
Needle driving: “Tubes”	23	76	56	85	70	82	73	75

In the procedural third part, fellows had to translate their improved theoretical and practical knowledge into daily robotic routine. In this context, the curriculum offered to perform hysterectomy and/or pelvic lymphadenectomy as moderate complex procedures.

Three months after initiation of part 3 of the curriculum all fellows had started robotic surgery in their home centers. Due to augmented knowledge in docking and assistance, they had become first or main assistant especially in complex, i.e. especially oncological procedures, which resulted in greater satisfaction at work. They had started to perform medium to complex procedures (e.g. benign hysterectomies with or without adnexectomy, myomectomy, adhesiolysis), mainly supervised. One candidate only started to perform the required lymphadenectomies in this first period.

In contrast to the outline of the Pilot Curriculum, regular assessment of progress in training was more done by open feedback than by use of GEARS/OSATS and NOTSS. Indeed, open feedback was usually deemed sufficient as tutors felt that they had a very structured and standardized way of operation, however, this was not per curriculum outlines.

Virtual procedural training was performed rarely due to limited access to simulators in the home educational center or as it was found too time-consuming in the real-life daily routine.

In the second half of the half year, in-house training proctoring of the candidates had been implemented in a more regular fashion in all home centers, while in some centers more than one supervisor was involved. Still, none of the fellows reported structural assessment by means of GEARS/OSATS and NOTSS, but with open feedback. Also, feedback was not yet given systematically after each procedure. Only one tutor followed the curriculum with structural assessment. Training was not done stepwise—even though standardized—per standards of the individual tutor. At the end of an approximately 10 months-in-house training all trainees could perform the index procedures unsupervised. There was a general plea for a more specified outline of the final curriculum, especially regarding the evaluation process of skills improvement.

For finalization of the program, fellows performed a hysterectomy (with or without adnexectomy) as index procedure and sent it to SERGS as a video for assessment using GEARS by each tutor. Summarized, scores show that finally all fellows performed the procedure without supervision achieving good or acceptable technical quality. Noticeably, the greatest difference can be seen in the parameter “efficiency” (see Table 2 and Supplementary Data, Tab. 4).

**Table 2:** Assessment of performance in index procedure at the end of training per GEARS-scale

Candidate	I	II	III	IV
Expert assigned from candidate	IV	I	II	III
GEARS				
Depth perception	3	5	4	4
Bimanual dexterity	4	4	5	4
Efficiency	2	5	4	2
Force sensitivity	3	5	5	3
Robotic control	3	4	5	3
Total	15	23	23	16

Formal approval of the completed logbook, results of the three didactic parts of the curriculum and of performance of the index procedure were revised by the SERGS Educational Committee and thereupon led to SERGS certification as robotic gynecological surgeon in this pilot curriculum.

More important than that, comments by fellows and tutors throughout implementation of the pilot curriculum were summarized to be included in a separate Delphi Process, whose results should improve the Curriculum on basis of a consensus on training guidelines for safe robot-assisted gynecological surgery.

## Discussion

Regarding the ever-expanding use of robot-assisted surgery in the last decade, several studies emphasize the benefit of both virtual and in vivo-training for proper surgical performance when using a robot. In a Pilot Study, the European Association of Urology showed feasibility of a 12-week structured training program for a definite procedure including virtual and mentored training in the operating room [15]. In accordance with existing literature, results of this multi-institutional study show feasibility of a tri-sectional educational program with a first part in didactics and virtual training, a second part with intensified training in the meaning of wet-lab courses and under guidance of an expert mentor, and a third part with supervised competency-based robotic surgery at dedicated education centers. Telephone video-conferencing proved useful for interim analysis of the curriculum, especially since structured training needs monitoring.

Our results show that the SERGS pilot curriculum is feasible and acceptable, as all fellows were content with their individual progress and their resulting status at their educational home institution. Definition of a standardized curriculum pushes both the qualification of the fellow and a further professionalism of the educational center that should fulfill educational criteria.

Fellows plead for more practical training, especially under supervision of an expert mentor. In this context, centralization of training in a fully equipped training center such as ORSI/Ghent was well perceived—which is in accordance with study results. [16].

Besides content of educational programs, there is discussion about the best instruments for evaluation of performance throughout. For virtual training, studies showed construct validity for evaluation of performance based on objective motion and time-based metrics [6]. These criteria are implemented in OSATS and GEARS. While these tools can be integrated in virtual simulators, assessment of clinical significance and behavior (NOTSS) is still the domain of a live supervisor. In our study, it proved hard for experienced tutors, used to open feedback, to adopt more validated and structured tools of assessment of technical and non-technical skills. Only in one case structured and systematic assessment was performed per the recommendations outlined in the curriculum. Tutors appeared particularly unfamiliar with competency-based assessment (as proposed in the CanMeds assessment [17, 18] such as by NOTSS. Train-the-trainers sessions seem necessary to become familiar with standardized evaluation tools. One supervisor video archived all procedures of his dedicated fellow, so that they were at least available for retrospective analysis. This concept could be an option to overcome the limits of time contingent available for the evaluation process—but it weakens the impact of a prompt feedback to the fellow.

Open feedback together with a less structured surgical training, as opposed to systematic structured assessment and modular training in index procedures, resulted in a longer than envisaged training period to gain sufficient competence. The ERUS programme clearly has shown that more systematic and stepwise training is more efficient and results in the shortest period to gain sufficient proficiency [15, 19, 20].

Assessment of performance in the hysterectomy video clips was finally done using GEARS systematic evaluation. This helped to standardize results for comparison and indicates that the curriculum has good educational impact and face validity: scores showed that finally all fellows could perform the procedure unsupervised with good or acceptable technical quality.

This study has some limitations: first the limited number of participants does not allow statistical analysis. Fellows were novices to console surgery but of different experience regarding their other gyneco-oncological and surgical competencies. Definition of an expert supervisor was not outlined in the curriculum, but all mentors assigned were high-volume robotic surgeons at teaching institutions. Exposure of training facilities was different among fellows: virtual simulators were not present at all institutions or were supplied throughout the course of the curriculum only.

## Conclusion

This study was planned as a Pilot Educational Program for modular robotic training of a defined common gynecological surgical procedure (i.e. robot-assisted laparoscopic hysterectomy) under supervision of experts. The tri-sectional modular structure with a didactic part and a simulator- and expert-mentored stepwise procedural training in the dry- and wet-lab setting, followed by supervised performance under real-life-conditions in the operating room, proves feasible, acceptable and with good educational impact. A structured and systematic training appeared to be difficult to implement, despite the proven efficiency of such an approach. Fellows and tutors need to be instructed and trained to adopt a more systematic training and assessment in surgical competencies.

Results from this first Pilot program can form the basis of a Delphi process, the objective of which is a consensus view of experts in terms of training guidelines for the safe introduction of robotic gynecological surgery.

## Electronic supplementary material

Below is the link to the electronic supplementary material.

Supplementary material 1 (PDF 172 kb)
