# Supplementary material for: The Society of European Robotic Gynaecological Surgery (SERGS) Pilot Curriculum for robot assisted gynecological surgery
Source: Arch Gynecol Obstet. 2017 Dec 13;297(2):415–20. doi: 10.1007/s00404-017-4612-5 (PMC5778155; doi:10.1007/s00404-017-4612-5)
Supplement: Supplementary file 1 — Supplementary material 1 (PDF 172 kb) [file 404_2017_4612_MOESM1_ESM.pdf]

The Society of European Robotic Gynaecological Surgery (SERGS) Pilot Curriculum for Robot Assisted Gynecological Surgery

Peter Rusch[a], Rainer Kimmig[a], Fabrice Lecuru[b], Jan Persson[c], Jordi Ponce[d], Michel Degueldre[e], René Verheijen[f]

[a]Department of Obstetrics and Gynaecology, University Hospital Duisburg-Essen, Essen, Germany

[b]Université Paris Descartes, Sorbonne Paris Cité, Faculté de Médecine, Paris, France
Assistance Publique-Hôpitaux de Paris, Hôpital Européen Georges-Pompidou, Chirurgie Cancérologique Gynécologique et du Sein, Paris, France

[c]Department of Obstetrics and Gynaecology, Skane University Hospital and Lund University, Lund, Sweden

[d]Department of Obstetrics and Gynaecology, Universitat da Barcelona, Barcelona, Spain

[e]European Robotic and Minimal Invasive Surgery Institute (ORSI) cvba, Melle, Belgium

[f]Department of Gynaecological Oncology, University Medical Center, Utrecht, Netherlands

Corresponding Author:
Peter Rusch

Department of Obstetrics and Gynaecology

University Hospital Duisburg-Essen

Hufelandstr. 55

45147 Essen, Germany

Email: peter.rusch@uk-essen.de

Phone: 0049-(0)201-723-85288

Fax: 0049-(0)201-723-5714)

ORCiD ID: 0000-0002-4890-3503

Suppl. Data: Tables

Requirement
existing dedicated robot assisted surgery team with at least one surgeon dedicated to the subspecialty of training
Committed and stable robot assisted surgery practice that is not under threat of major changes during the period of training
existing operation policy, procedure guidelines and treatment protocols for robot assisted surgery relevant to the training program
Clear policy of training the trainers portfolio
Offer the opportunity of cross training and experience such as having an ongoing robot assisted surgery program/practice in colorectal and urologic surgery
adequate workload in robot assisted gynecological surgery in chosen area of training, for example<br>i. >300 robotic surgery cases/year<br>ii. >50 robotic gynecological surgery cases/year<br>iii. >20 robot assisted oncological surgical cases per subspecialty/year<br>iv. >20 robotic gynecological surgery cases per surgeon/year
Mature Clinical Governance portfolio with minimum requirements:<br>i. ongoing audits of perioperative characteristics<br>1. total operative time<br>2. blood transfusion rate<br>3. conversion to laparotomy rate<br>4. perioperative complications: type and rate<br>5. length of hospital stay<br>ii. ongoing audits of programme efficiency reflecting financial accountability:<br>1. theatre utilization profile<br>2. length of waiting list as compared to previous performance<br>3. above selected audits as blood transfusion, conversion rates and lengths of hospital stay<br>iii. regular risk management and morbidity/mortality meetings to discuss relevant incidents

Tab.1: Criteria of eligibility as Center of Excellence for participation in SERGS-Pilot-Curriculum

**First Period**<br>Basic Training: orientation&models			
Type of training	Training part	Type of assessment	Modality of verification
Didactic	Knowledge of system<br>Knowledge of procedures	Exam	Online module (manufacturer) or Supervisor
Dry skills	Use of console & instruments<br>Set-up of robot<br>Solving common (technical) problems	Participation	Supervisor
	Practice validated skills	Baseline Skills Evaluation (5Excercises/Exc.) (1=poor/5=excellent)<br>#1: Robotic docking and instrument insertion<br>#2: RingRollercoaster1<br>#3: RingRollercoaster2<br>#4: RingRollercoaster3<br>#5: RingRollercoaster4	Supervisor<br><br>Exc.#2-5 substitutable by DaVinci Mimic Skills Simulator (dV) (if available)
Virtual	Learning the system	Participation	Online module (manufacturer) or Supervisor
	Practice validated skills	Test (7Exc.)<br>#6: Camera&Clutching<br>#7: Endowrist Manipulation<br>#8: Energy&Dissection<br>#9: thread the rings<br>#10: suture sponge<br>#11: dots&needles<br>#12: interrupted suturing	DaVinci Mimic Skills Simulator (if available)
**Second Period**<br>Hands-on training (ORSI)			
Didactic	Knowledge of system<br>Knowledge of procedures	Participation	ORSI-supervisor
Virtual	Practice procedural skills<br>View/life surgery	Test (dV)<br>Participation	ORSI-supervisor
Animal	Practice basic skills<br>Practice hysterectomy<br>Practice lymphadenectomy	BSE/GEARS<br>NOTSS/OSATS<br>NOTSS/OSATS	ORSI-supervisor
**Third Period**<br>In-house, mentored training			
Didactic	Indciations&types of surgery	Exam	In-house-supervisor
Virtual	Practice procedural skills	Test	dVMimic Skills Simulator
Bedside	Stepwise hysterectomy<br>Stepwise lymphadenectomy	NOTSS/OSATS	In-house supervisor
	Perioperative care	Participation	In-house supervisor
	Video documentation of stepwise procedure	GEARS	External supervisor

Tab. 2: Trisectional course of SERGS Pilot Curriculum (adapted from Schreuder et al. 2012)

**Candidate**	**No.1**	**No.2**	**No.3**	**No.4**
**Origin**	Spain	France	Sweden	Germany
**Age**	32years	30years	42years	39years
**Center of Excellence**	Hospital Belvitge, Barcelona	Europ. Hospital Pompidou, Paris	Skånes University Lund	University of Duisburg-Essen
**Subspecialty** (up-to-date)	Oncology, Minimal Invasive Surgery, Open Surgery	Oncology, Minimal Invasive Surgery, Open Surgery	Oncology, Minimal Invasive Surgery (i.e. Endometriosis)	Oncology, Breast Surgery, Minimal Invasive Surgery

Tab. 3: Characteristics of fellows and educational institution

Depth perception				
1	2	3	4	5
Constantly overshoots target, wide swings, slow to correct		Some overshooting or missing of target, but quick to correct		Accurately directs instruments in the correct plane to target
**Bimanual dexterity**				
1	2	3	4	5
Uses only one hand, ignores nondominant hand, poor coordination		Uses both hands, but does not optimize interaction between hands		Expertly uses both hands in a complementary way to provide best exposure
**Efficiency**				
1	2	3	4	5
Inefficient efforts; many uncertain movements; constantly changing focus or persisting without progress		Slow, but planned movements are reasonably organized		Confident, efficient and safe conduct, maintains focus on task, fluid progression
**Force sensitivity**				
1	2	3	4	5
Rough moves, tears tissue, injures nearby structures, poor control, frequent suture breakage		Handles tissues reasonably well, minor trauma to adjacent tissue, rare suture breakage		Applies appropriate tension, negligible injury to adjacent structures, no suture breakage
**Autonomy**				
1	2	3	4	5
Unable to complete entire task, even with verbal guidance		Able to complete task safely with moderate guidance		Able to complete task independently without prompting
**Robotic control**				
1	2	3	4	5
Consistently does not optimize view, hand position, or repeated collisions even with guidance		View is sometimes not optimal. Occasionally needs to relocate arms. Occasional collisions and obstruction of assistant.		Controls camera and hand position optimally and independently. Minimal collisions or obstruction of assistant

Tab. 4: Global Evaluative Assessment of Robotic Skills (GEARS) (adapted from [1]): a rating of 1 reflects the lowest level of performance while a rating of 5 is considered the highest proficiency. An overall performance score is derived by summing the ratings in each domain.

References

[1] Goh AC, Goldfarb DW, Sander JC, Miles BJ, Dunkin BJ. Global evaluative assessment of robotic skills: validation of a clinical assessment tool to measure robotic surgical skills. J Urol 2012; 187(1): 247–52 [https://doi.org/10.1016/j.juro.2011.09.032][PMID: 22099993]
